# Meningococcal vaccine introduction in Mali through mass campaigns and its impact on the health system

**DOI:** 10.9745/GHSP-D-13-00130

**Published:** 2014-01-15

**Authors:** Sandra Mounier-Jack, Helen Elizabeth Denise Burchett, Ulla Kou Griffiths, Mamadou Konate, Kassibo Sira Diarra

**Affiliations:** a London School of Hygiene & Tropical Medicine, London, UK; b Independent Consultant, Bamako, Mali

## Abstract

**Objective:**

To evaluate the impact of the meningococcal A (MenA) vaccine introduction in Mali through mass campaigns on the routine immunization program and the wider health system.

**Methods:**

We used a mixed-methods case-study design, combining semi-structured interviews with 31 key informants, a survey among 18 health facilities, and analysis of routine health facility data on number of routine vaccinations and antenatal consultations before, during, and after the MenA vaccine campaign in December 2010. Survey and interview data were collected at the national level and in 2 regions in July and August 2011, with additional interviews in January 2012.

**Findings:**

Many health system functions were not affected—either positively or negatively—by the MenA vaccine introduction. The majority of effects were felt on the immunization program. Benefits included strengthened communication and social mobilization, surveillance, and provider skills. Drawbacks included the interruption of routine vaccination services in the majority of health facilities surveyed (67%). The average daily number of children receiving routine vaccinations was 79% to 87% lower during the 10-day campaign period than during other periods of the month. Antenatal care consultations were also reduced during the campaign period by 10% to 15%. Key informants argued that, with an average of 14 campaigns per year, mass campaigns would have a substantial cumulative negative effect on routine health services. Many also argued that the MenA campaign missed potential opportunities for health systems strengthening because integration with other health services was lacking.

**Conclusion:**

The MenA vaccine introduction interrupted routine vaccination and other health services. When introducing a new vaccine through a campaign, coverage of routine health services should be monitored alongside campaign vaccine coverage to highlight where and how long services are disrupted and to mitigate risks to routine services.

## Abstract (precis)

The meningococcal A vaccine campaign led to major disruption of routine vaccination services and reduced other services, notably antenatal care.

## INTRODUCTION

As countries introduce new vaccines at an ever-increasing pace, there have been concerns about the effect on immunization programs and health systems.^1^ There is limited evidence, however, on the effects of introducing new vaccines on health systems. A recent review found this was rarely the main focus of studies and that research seldom focused on low-income countries, such as Mali, where health systems are generally weaker.^2^

Broader studies have shown that vertical disease control programs can have both positive and negative effects on the wider health system.^3^^–^^7^ Such findings have led to regular calls for a more integrated approach to implementing communicable disease programs.^4^^,^^8^^–^^13^

New vaccines may be introduced into the health system through a variety of strategies. They can be added to the routine immunization schedule (delivered in health facilities or through routine outreach), through targeted mass campaigns, or a combination of both.

Starting at the end of 2010, the meningococcal A (MenA) vaccine (MenAfriVac) has been introduced through phased mass campaigns in the high-prevalence meningococcal belt in West Africa.^14^^,^^15^ (MenAfriVac presents in a 10-dose lyophilized vial and needs to be preserved at temperatures between 2°C–8°C. Once open, a vial should be used within 6 hours.) Several studies have scrutinized vaccination campaigns, some of them pointing to a possible disruption of routine services.^16^^–^^19^

Between September 2010 and November 2011, Mali introduced the MenA vaccine over 3 separate campaigns. The first pilot phase occurred in 2 districts, followed by a second phase in Bamako, Ségou, and Koulikoro regions in December 2010. In November 2011, the third phase covered the remaining 6 regions. The campaigns targeted all people 1–29 years old, and each campaign ran for 10 days, vaccinating a total of 10 million people. Vaccination took place at health facilities and through outreach in community settings, in both rural and urban areas.

Between 2010 and 2011, Mali vaccinated 10 million people with the meningococcal A vaccine through 3 campaigns.

### The Health Setting in Mali

Mali, a low-income country in West Africa, has a population of 14.85 million (Table 1). It has a young population, with 46% of the country's population under 15 years old.

**TABLE 1.** Mali Country Profile, 2012

**Key Indicators**		**Source**
Population	14.85 million	World Bank^20^
Life expectancy at birth	54 years	World Bank^20^
Under-5 mortality rate	176/1,000 live births	WHO^21^
Maternal mortality rate	464/100,000 live births	Ministry of Health (Mali)^22^
Fertility rate	6.6 children/woman	Ministry of Health (Mali)^22^
Population living in rural areas	70%	Ministry of Health (Mali)^22^
DTP3 coverage rate (national)	74%	WHO^21^
GNI per capita	US$660	World Bank^20^

Abbreviations: DTP3, 3rd dose of diphtheria-tetanus-pertussis vaccine; GNI, gross national income; WHO, World Health Organization.

Health care is delivered through community-owned health facilities (*Centres de Santé Communautaires*, also called CSCOM). Key health care professionals, such as head doctors and nurses, are usually employed by the government while other staff and operational costs are supported by the local community. Vaccination is provided free of charge, but user fees are charged for most other services.

In Mali, the Expanded Programme on Immunization (EPI) delivers 10 different antigens to children under 5 years old, through a combination of fixed immunization sessions at health facilities, outreach services to community sites, and campaigns. In 2011, the vaccination coverage rate of the third dose of diphtheria-tetanus-pertussis (DTP3) vaccine was 74%.^21^

Many health interventions in Mali are delivered through campaigns, rather than through routine services. During 2010 and 2011, 28 health campaigns were conducted, totaling 134 days (Table 2). Twenty-one were national campaigns and were used to deliver a range of interventions, such as vaccinations, insecticide-treated bed nets, deworming drugs, and nutritional supplements; 6 had a regional focus; and 1 was conducted in only 2 districts (Table 2). The campaigns typically lasted 2–10 days, with an average of 1 campaign every 3 weeks.

**TABLE 2.** Public Health Campaigns in Mali, 2010–2011

**Campaign**	**Geographic Focus^a^**	**Dates**
**2010**		
Polio NID (Sikasso region), 1st round	1 Region	Mar 6–9
Polio NID (Sikasso region), 2nd round	1 Region	Mar 26–28
Polio NID, 1st round	National	Apr 24–27
Polio NID, 1st round	National	May 28–31
NTDs (distribution of ivermectin and albendazole)	National	Jun 14–16
NTDs (distribution of praziquantel and azithromycin)	National	Jun 18–20
Polio NID, 2nd round	National	Jun 25–28
MILDA (distribution of bed nets)	National	Jul 17–20
Preventative campaign for severe malnutrition	National	Sep 16–18
**MenAfriVac**, 1st phase	**2 Districts**	Sep 13–20
Polio NID, 3rd round	National	Oct 28–31
Polio NID, 3rd round	National	Nov 25–27
National Week of Nutrition (SIAN)	National	Dec 1–7
**MenAfriVac**, 2nd phase, Koulikoro, Ségou, and Bamako regions	**3 Regions**	Dec 14–23
**2011**		
Measles SIA	National	Feb 28–Mar 6
Polio NID, 1st round	National	Mar 25–28
Polio NID, 2nd round	National	Apr 29–May 2
Polio NID, 3rd round	National	Jun 3–6
National Week of Nutrition (SIAN)	National	Jun 4–10
Polio NID, 4th round	National	Jun 23–26
NTDs (distribution of ivermectin and albendazole)	National	Jul 14–19
NTDs (distribution of praziquantel and azithromycin)	National	Jul 27–Aug 1
Polio NID	4 Regions	Jul 29–Aug 1
Polio NID	5 Regions	Aug 20–23
Polio NID, 4th round	National	Sep 30–Oct 3
Polio NID, 5th round	National	Oct 28–31
**MenAfriVac**, 3rd phase, other regions	**6 Regions**	Nov 15–24
Polio NID, 6th round	National	Nov 26–29

Abbreviations: MILDA, moustiquaire imprégnée d'insecticide à longue durée d'action (long-lasting insecticidal net); NID, National Immunization Days; NTDs, neglected tropical diseases; SIA, supplementary immunization activities; SIAN, Semaine d'Intensification des Activités de Nutrition (Child Nutrition Week).

Data from the Expanded Programme on Immunization (EPI) of Mali.

a Mali has 9 regions.

This study aimed to evaluate the impact of introducing the MenA vaccine on the routine immunization program and the wider health system in Mali during the second phase of the MenA vaccine introduction (in 3 regions). It was part of a larger study that explored the impact of a range of new vaccine introductions in 6 low- and middle-income countries.^23^

## METHODS

We used a mixed-methods study design, combining semi-structured interviews with stakeholders, a health facility survey, and analysis of routine health facility data. Fieldwork was conducted in July and August 2011, with additional national stakeholders interviewed in January 2012.

Data were collected at the national level and in 2 of the 3 regions (Bamako and Koulikoro) in which the second phase of the MenA vaccination campaign took place. In both regions, 3 districts were purposively selected to reflect different ranges of vaccination coverage and profiles of urbanization/rurality.

### Conceptual Framework

Our analytical framework, developed by the World Health Organization (WHO) ad-hoc working group on new vaccines and health systems, was an adapted version of the WHO Health Systems Framework.^1^^,^^24^ It consisted of the same 6 “building blocks” from the WHO Health Systems Framework but with the addition of vaccination-specific elements within these (Table 3).

**TABLE 3.** Framework for Assessing the Health Systems Impact of New Vaccine Introduction

**Health System Building Block**	**Examples of Vaccination-Specific Elements**
Service delivery	Demand and acceptance Access and utilization Quality of care Delivery modalities
Health workforce	Availability and distribution of staff Training and capacity of staff Remuneration and satisfaction Performance and supervision
Health information system	Routine data collection and reporting Disease surveillance
Medical products, vaccines, and technologies	Forecasting of vaccines and injection supplies Procurement and stock management Cold chain management and waste disposal
Financing and sustainability	Affordability Domestic financing External financing
Leadership/governance	Regulatory policy Political commitment Organization, structure, reform, negotiation, stewardship

Source: WHO Ad-hoc Working Group on Impact of New Vaccines on Health Systems^25^

### Stakeholder Interviews

Thirty-one stakeholders at national, regional, and district levels were interviewed (Table 4). Most interviewees were purposively selected because of their involvement in the vaccine introduction process. In addition, stakeholders with responsibilities outside the EPI were interviewed to seek their perception of the impact on the broader health system and to explore the extent to which they had collaborated with those involved in the campaign. Interview questions aimed to investigate critical aspects of each of the 6 building blocks that may have been affected either positively or negatively by the new vaccine introduction. They also aimed to understand how various stakeholders were involved in the introduction process.

**TABLE 4.** Number and Types of Key Informants, by Health System Level

**Health System Level and Type of Respondent**	**Data Collection Method**	**Number**
National		
EPI, MOH	Interview	2
Other, MOH	Interview	8
Civil society and international agencies	Interview	5
Academics/other domestic agencies	Interview	4
Regional		
Regional head doctor	Interview	3
District		
District head doctor and other staff involved in vaccination activities	Interview	9
Facility		
Health facility staff	Questionnaire	18
**Total**		**49**

Abbreviations: EPI, Expanded Programme on Immunization; MOH, Ministry of Health.

### Health Facility Surveys

In each district, 3 health facilities were selected based on increasing distance from the district's main urban center. The aim was to survey a variety of urban, semi-rural, and remote health care facilities in each district. Staff from 18 health facilities were surveyed using a researcher-administered questionnaire (Table 4), adapted from the Post-Introduction Evaluation (PIE) methodology used in the vaccination field.^26^

### Routine Health Facility Data

Routine data were collected on the number of children vaccinated and antenatal care (ANC) visits per day during the campaign and one month before and after, in order to explore the continuity of health services during the campaign.

### Data Collection and Analysis

Prior to administering the interviews and facility questionnaires, the aim of the study was explained to participants and an information sheet was provided. After discussing any questions or concerns, participants signed a consent form. Where permitted, interviews were recorded and transcribed. When they were not recorded, notes were taken and typed up in detail afterwards. Survey responses were recorded directly onto the paper questionnaires. The interviews and surveys were conducted in French.

Framework analysis was used to explore the interview data.^27^ An initial coding framework was developed based on preliminary assessment of the transcripts and the building-blocks framework. These codes were applied to all the interview transcripts, and the data within each code were then explored for themes and patterns. The software Open Code was used to manage the data.^28^ Survey data were entered into SPSS and analyzed using descriptive statistics.

Ethical approval was obtained in Mali and from the London School of Hygiene & Tropical Medicine.

## FINDINGS

The surveyed facilities had between 2 and 16 staff members. In their normal practice outside campaigns, almost all the facilities reported having 1 or 2 routine vaccination sessions per week, with 2 of 18 facilities holding sessions only every 4 weeks.

The introduction of the MenA vaccine had no effect (either positive or negative) on many aspects of the health system. Some aspects did improve, however, while others suffered. The majority of effects were felt on the EPI, rather than on the broader health system. The findings are presented below according to the 6 building blocks.

### Service Delivery

#### High Demand and Acceptance

Interviewees and health facility staff universally reported that there was high demand for the new vaccine. Social mobilization raised awareness about the vaccine, and rumors were well-managed. Because of its large reach, respondents felt that the MenA vaccine campaign had improved awareness of the benefits of vaccination and had increased credibility of the EPI.

#### Reduced Access to and Use of Routine Services

Of the 18 surveyed health facilities, 12 had either no record of any routine vaccination activities during the 10-day MenA vaccine campaign or, when records were not available, stated they had stopped routine vaccination (Table 5). In the Koulikoro region, only 2 of 9 health centers (22%) provided routine vaccination services during the campaign. Among the 6 facilities in the 2 regions that continued routine vaccination services, 1 facility vaccinated only 1 child while another continued as usual only because Médecins sans Frontières (MSF) staff had undertaken the MenA vaccine campaign independently in the Kati District. However, even though MSF carried out the campaign, staff from 2 of the surveyed health centers in the Kati District were sent on supervision duties, which affected routine activities.

**TABLE 5.** Continuity of Routine Vaccination Activities at Health Facilities During Meningococcal A Vaccine Campaign (N = 18 Facilities)

**Region**	**No. of Facilities**	
	**Discontinued Routine Vaccination**	**Continued Routine Vaccination**
Bamako	5	4
Koulikoro	7^a^	2
**Total**	**12**	**6**

a In 3 of the 7 facilities, data were based on health facility staff recall because records of routine vaccination activities were not available.

Likewise, during the campaign, routine outreach vaccination services were discontinued in 5 facilities, while 2 facilities with infrequent outreach services were able to accommodate or postpone the work. (The remaining facilities did not conduct outreach.) Many interviewees, notably at national and regional levels, stated that routine vaccination had been maintained during the campaign, but findings from the facility survey and routine data showed that this was not the case. The number of staff per health center did not seem to affect whether vaccination services continued, as the median number of staff did not differ between those facilities that continued vaccination services and those that did not.

Routine clinic and outreach services were discontinued during the MenA campaign in most facilities.

Data collected from health facilities before, during, and after the MenA vaccine campaign suggest that the campaign had considerable impact on routine vaccination activities and, to some extent, on ANC consultations. The average daily number of children vaccinated during routine services was 79% lower during the 10-day campaign period in December 2010 than during the first 13 days of the month before the campaign started, and 87% lower than during the last 8 days of the month when the campaign was over (Table 6). While fewer children were vaccinated per day during the mid-period of all 3 months (14th through 23rd), the average number was 71% and 74% less during the campaign days than during similar days in November and January, respectively. Antenatal care consultations also decreased during the campaign but not as severely—there were 10% and 15% fewer consultations compared with the same days of the month during November and January, respectively.

**TABLE 6.** Average Daily Number^a^ of Routine Services Before, During, and After the MenA Vaccination Campaign

**Days of the Month**	**Nov 2010**	**Dec 2010**	**Jan 2011**
**Average Daily No. of Children Vaccinated**^b^ **(in 15 health facilities)**			
1–13	82	62	62
**14–23**	45	**13**	50
24–30/31	70	99	81
**Average Daily No. of ANC Consultations (in 18 health facilities)**			
1–13	88	91	90
**14–23**	63	**56**	66
24–30/31	90	94	121

Abbreviations: ANC, antental care; MenA, meningococcal A.

Cells in boldface pertain to the MenA vaccine campaign period (December 14–23, 2010).

a Only working days were considered in the calculation of the average daily service activity to make comparisons between months meaningful.

b Children receiving routine vaccinations.

There was less impact on the delivery of other health services, but nonetheless one-third of facilities (6 of 18) reported a reduction in services, and 2 facilities closed their ANC services for the duration of the campaign. Most of the facilities that reported a reduction in ANC and outpatient services were situated in the Koulikoro region where staffing levels were lower than in Bamako.

At health facility level, there is an impact [on other activities] because these have only 2 staff in most cases … the workload is much increased and some activities are reduced because all the staff are involved [in the campaign].

–District official, Koulikoro

Many interviewees noted the regular discontinuation of routine services for a range of health and immunization interventions during the numerous campaigns that were organized throughout the year. Some interviewees criticized the frequency of campaigns; others underlined their negative impact on the health system.

It [the effect of the campaign on health services] is immaterial [that is, difficult to measure] but this is significant; it is an issue that can lead to loss of trust in the service.

–National Ministry of Health (MOH)

Two interviewees commented that the new vaccine improved equity, notably for specific groups for which meningitis would pose a greater risk, such as people affected by sickle cell anemia.

#### Vertical Delivery Modality

The campaign was organized similarly to other vaccination campaigns, with a combination of fixed posts and outreach teams. It did not involve any co-delivery of other interventions (for example, bed nets, deworming), nor did it involve catch-up of defaulters for other vaccines. Furthermore, once over, the campaign did not lead to a deliberate change in how either the EPI or other health services were delivered. The majority of interviewees and some facility staff felt that this was a missed opportunity. Some interviewees suggested that a more integrated approach with other services might have been positive while others commented that the high resulting workload would have made co-delivery of other services impossible.

The MenA campaign did not deliver other health services to people, nor did it provide missed vaccinations for other vaccines.

Many people come during the campaign, and this would be a unique opportunity to do more interpersonal communication and to raise awareness on vaccination.

–District official, Bamako

We would like to use the vaccination campaign to provide other services for children such as vitamin A and deworming … it only happened once in 2007 during a measles campaign. We need to provide a more integrated service and the spirit of integration is not always shared.

–National MOH

Considering the workload, if we combined the MenA campaign with other services, that would not work.

–District official, Koulikoro

### Health Workforce

#### Increased Workload

Additional staff members were called upon for the duration of the MenA vaccination campaign, and workload was reported to be high during the period. Staff numbers and workload returned to normal levels once the campaign ended. It was mentioned that the workload impact was substantial in remote regions.

If in the North you have 3 persons, these will do only the campaign during 10 days.

–National MOH

#### Enhanced Training and Capacity of Staff

National and sub-national health service managers overwhelmingly stated that training for the MenA vaccine introduction had enhanced provider skills, often beyond those required for the specific vaccine, acting as a general refresher on vaccination skills. They commented that a particular emphasis had been placed on adverse events following immunization (AEFI), waste management, surveillance, and social mobilization.

Training for the MenA vaccine served as a general refresher on vaccination skills for providers.

This is thanks to MenAfriVac that I received a theoretical training for the first time, although I had been working in EPI since 2008.

–District official, Bamako

It was quite a comprehensive training … a training like that is always useful, unlike for polio for which we have no more training, and people tend to do a bad job.

–Regional official, Bamako

However, a large majority of health facility staff (15 of 18) reported that training had focused primarily on the new vaccine, possibly reflecting the fact that the new vaccine was a “one-off campaign” and was not yet introduced into the routine schedule.

#### Motivated Staff

Interviewees generally felt that the introduction of the MenA vaccine had motivated staff, because of the expected outcome of reduced child morbidity and mortality, or because the campaign was associated with the payment of a per diem. The per diem received ranged from 2,500 to 5,000 CFA Francs (approximately US$5 to $10) per day.

During the campaign, staff is motivated, unlike during the routine [service period] when there is no financial motivation.

–District official, Bamako

### Health Information Systems

All respondents reported that there were no fundamental changes to the health information system as a result of introducing the new vaccine. However, a strong emphasis was placed on AEFI surveillance during the introduction itself, including strengthening the skills of health workers to recognize and report AEFI and training of laboratory staff. Despite perceived better awareness, interviewees at the national and regional level acknowledged that AEFIs were still not routinely reported.

### Medical Products, Vaccines, and Technologies

Interviewees explained that in 2010, prior to the introduction of the MenA vaccine and another new vaccine (pneumococcal conjugate vaccine), the cold chain was strengthened in 4 regions (including our study regions) and 1,050 vaccine carriers were provided to health facilities. Overall, interviewees reported that the cold chain capacity was sufficient to handle the MenA vaccination campaign. They explained that this was because routine vaccination had been discontinued, routine vaccine stocks were temporarily relocated to alternative (regional and district) places, and other temporary cold storage was used.

Two of our fridges were full with the MenAfriVac vaccines, so our routine vaccines were stored at regional level and that was not an issue because we did not do any routine vaccination apart from BCG [bacille Calmette-Guérin] and polio birth doses. This is similar during the measles campaign but not for polio, as in general we do not stop routine vaccination.

–District official, Bamako

The introduction of the MenA vaccine had no impact on forecasting and procurement. It had some limited positive impact on waste disposal, with reports of several upgrades to waste management equipment. However, one district noted a major delay in disposing of a large amount of injecting materials.

### Financing and Sustainability

The introduction of the MenA vaccine was mainly funded by external partners, such as the Global Alliance for Vaccines and Immunisation (GAVI), WHO, the United Nations Children's Fund (UNICEF), and the Canadian International Development Agency (CIDA), although the government of Mali also committed funds. There was no indication that resources for the new vaccine had displaced other investments, although several interviewees noted that funding was easier to commit for vaccination than for many other health services.

The total cost of the campaign amounted to US$9.4 million, according to the EPI.^29^ A few interviewees suggested that the introduction of 2 new vaccines (the MenA vaccine and the pneumococcal conjugate vaccine) in 2011 could have negatively impacted overall EPI operational costs.

A major positive effect noted by both interviewees and facility staff was the expected sharp decrease in meningococcal disease that enabled a shift from epidemic response to prevention. This was considered beneficial and cost-effective for the health system.

The meningitis outbreak response in Bamako in 2009 lasted over 10 days and had to mobilize a lot of resources and a high number of vaccinators.

–National MOH

The majority of interviewees and respondents at facilities felt that the funds planned for the implementation of the campaign were sufficient to cover its costs. Only a few interviewees commented that additional funds drawn from the routine budget were used to pay for transport, fuel, and communication costs and, in one district, to supplement staff per diems for implementing the campaign.

Interviewees and some facility staff reported that the cancellation or reduction of routine activities during the campaign had, in some cases, caused a reduction in fee-for-service revenues.

Reduction of routine services, which usually involve user fees, during the MenA campaign sometimes resulted in reduced facility revenue.

Campaigns reduce routine activities because staff deserts the facility. On one hand, this has an impact on financial revenues of the facility, and on the other hand, it benefits staff through per diem. That is why staff do not usually complain about campaigns.

–National MOH

### Leadership and Governance

Interviewees noted that the government had demonstrated high political commitment to the vaccine introduction and that some aspects of governance were strengthened. For instance, AEFI surveillance was developed through the activation of national and sub-national committees on AEFI. Capacities were further enhanced through locally conducted clinical trials to assess the safety and efficacy of the new vaccine and participation in multicountry studies.^30^^,^^31^

Regulatory norms and standards were updated in preparation for the introduction of the MenA vaccine, including guidelines and training modules. However, regulatory approval for MenAfriVac was bypassed because of time pressure.

Collaboration among the National Communication Agency for Health, the Centre for Vaccine Development, and the Ministry of Health was established or strengthened during the preparatory phases of the introduction, which was judged to have long-term positive effects. This resulted in the use of scientific evidence, including formative research, for communication, which benefited the broader EPI communication strategy and helped to focus on aspects such as safety and preventing rumors. Multidisciplinary teams for hospital surveillance studies were also established. However, enhanced collaboration did not occur between departments within the Ministry of Health; there was no involvement of other service delivery departments in planning for the campaign and no discussion of co-delivering other interventions with the new vaccine.

Collaboration between health agencies improved during the MenA campaign but not between departments within the Ministry of Health.

Usually everybody remains in their own silo … for instance, before [the MenA vaccine introduction] we were not interested in post-marketing surveillance … but this is key for effective communication.

–National stakeholder

I wish we would have been associated with the new vaccine activities … the spirit of integration is not always well-understood because each service thinks they can achieve their results separately.

–National MOH

## DISCUSSION

Our study is the first to triangulate findings about stakeholders' perceptions of the effect of introducing the new MenA vaccine on health services with routine health facility data. The study found that many aspects of the health system were not affected by the MenA vaccine introduction, either positively or negatively, while some aspects improved and others—notably, continuity of routine services—suffered.

### Positive Effects of the Vaccine Campaign

Some of the perceived positive effects of the new vaccine introduction on the routine immunization program included improved governance and collaboration, communication and social mobilization, surveillance, and provider skill strengthening (particularly those relevant to EPI, such as AEFI surveillance and waste management). In addition, a separate evaluation of the campaign showed high recognition of the disease, with 80% of people in the community surveyed knowing the disease prevented by the new vaccine.^32^ Furthermore, a widely perceived positive effect was the reduction of disease and, to a lesser extent, recognition that equity had been improved at the population level. The MenA vaccination also led to expectations of future resource savings and service planning improvements due to the avoidance of costly outbreak responses and reactive vaccination campaigns.

### Routine Services Reduced Due to Vaccine Campaign

The main negative effect identified by the interviewees was the discontinuation or sharp reduction in routine vaccination services and, to a lesser extent, reduction in the availability of other health services, such as ANC, during the 10 days of the campaign. Analysis of routine health facility data confirmed this finding. It is worth noting that some interviewees believed that routine vaccination continued as usual during the campaign, but this was not confirmed by routine data. A recent quantitative study carried out in South Africa found a similar association between measles campaigns and decreased routine immunization coverage.^33^ There is a risk that the MenA vaccine introduction may have had a negative effect on routine vaccination coverage due to the disruption in routine services.

However, some interviewees argued that a countrywide vaccination campaign using the new conjugate MenA vaccine would prevent repeated, reactive epidemic outbreaks that also interrupt routine health services. For example, in its 2007 outbreak response, Burkina Faso spent US$6.08 million (3% of the health care budget) on vaccinating 4.1 million people plus another US$3 million on meningitis case management. It is worth noting that because of the short duration of protection provided by the polysaccharide vaccine used during this outbreak response, such costs would occur regularly.^34^

The effects of the MenA vaccine campaign on routine health services should be examined in the broader context in which as many as 14 vaccination campaigns occur each year. Although the effect of an individual campaign might seem limited in the short term, the impact of multiple campaigns is cumulative. In fact, interviewees stressed that the high frequency of campaigns had an adverse effect on the availability and credibility of routine health services. Previous studies have described drawbacks and benefits of mass vaccination campaigns,^18^^,^^35^^,^^36^ and they have pointed out that countries with weaker health systems are more likely to experience adverse effects of campaigns, such as disruptions to health services.^17^^,^^37^^,^^38^

The impact of multiple vaccination campaigns each year on routine health service delivery can add up.

In line with a recent study, we found there was a tension between the personal financial benefit (per diems) received by staff during the campaign and the recognition that the campaign may disrupt health services.^39^

### Lack of Integration of Vaccination Campaign With Other Services

As far as the authors are aware, no other study has yet reported on the effects of new vaccine introductions by mass campaign on health systems.^2^ Overall, the meningococcal vaccine introduction in Mali was implemented in a conventional vertical manner. The introduction did establish some valuable collaborations, but it was generally not used as a means to strengthen the health system. Our study provides some evidence that the mass-campaign delivery mode may disrupt the provision of routine services and reduce fee-for-service revenues linked to routine medical services, although our methodology was not designed to answer the latter. This aspect needs to be investigated further, particularly in terms of the possible impact on those routine activities traditionally financed using fee-for-service revenues.

The MenA vaccine campaign in Mali was successfully resourced, planned, and delivered, and it achieved high coverage.^32^ Mass campaigns are generally considered an effective means of achieving high coverage rates.^40^ However, the reach to a large target population—and to subgroups, such as young people, that seldom use health services—was not used to provide other benefits or services to this population, particularly in more remote regions where contact with health services is rare. We found that this was likely to be caused by insufficient coordination between departments within the Ministry of Health and because of earmarked funding for the vaccination campaign.

The lack of consideration of a more integrated approach and the lack of involvement of other partners in the planning process were seen by some as a missed opportunity. Strong tension remains between the ambition to achieve expected coverage in order to prevent epidemic outbreaks and the opportunity to use the campaign to bring other services to the population, such as health promotion or nutrition. Both perspectives were cited by respondents in our study. Delivering other interventions during the MenA vaccine campaign would have involved important challenges (such as logistical, financial, and communication), as other researchers have also noted.^41^^–^^43^ However, the overall lack of discussion at the level of the Ministry of Health for such activities meant that the campaign remained very vertical. Other researchers have also noted that there are many missed opportunities for using campaigns to strengthen integrated service delivery.^37^^,^^38^^,^^41^^,^^44^

### Relevance of Findings to Other Vaccine Initiatives

The MenA vaccine introduction may be seen as having unique features that are not typical of either a routine vaccine introduction or a regular supplementary immunization activity. However, it does have broader relevance given that other new vaccines in the next few years are likely to follow the MenA vaccine introduction model of starting with a catch-up campaign before introducing the vaccine into the routine schedule. This could be the case with human papillomavirus (HPV), Japanese encephalitis, and measles-mumps-rubella vaccines.

## CONCLUSION

Recommendations to promote health systems strengthening with vertical disease control activities are not new.^16^^,^^19^ However, as long as financing remains vertical, with each program setting its own specific objectives, it will be difficult to mitigate the adverse effects of vaccine campaigns on routine services. If countries and international partners truly want to promote health systems strengthening with new vaccine introductions, they should start by measuring not only vaccine campaign target coverage but also its effect on the use of routine health services. This would enable EPI staff to take stock of existing disruptions, adapt planning, and reallocate resources to ensure that routine services continue to be delivered during campaigns.
